# Copper(II) partially protects three histidine residues and the N‐terminus of amyloid‐β peptide from diethyl pyrocarbonate (DEPC) modification

**DOI:** 10.1002/2211-5463.12857

**Published:** 2020-04-29

**Authors:** Merlin Friedemann, Vello Tõugu, Peep Palumaa

**Affiliations:** ^1^ Department of Chemistry and Biotechnology Tallinn University of Technology Estonia

**Keywords:** amyloid‐beta peptide, Cu(II) ions, diethyl pyrocarbonate, ESI Q‐TOF MS, insulin, MALDI‐TOF MS, MS

## Abstract

Diethyl pyrocarbonate (DEPC) has been primarily used as a residue‐specific modifying agent to study the role of His residues in peptide/protein and enzyme function; however, its action is not specific, and several other residues can also be modified. In the current study, we monitored the reaction of DEPC with amyloid‐beta (Aβ) peptides and insulin by matrix‐assisted laser desorption/ionization time‐of‐flight mass spectrometry (MALDI‐TOF MS) and determined the modification sites by electrospray ionization quadrupole time‐of‐flight tandem MS (ESI Q‐TOF MS/MS). Our results indicate that five residues in Aβ1–42 are modified in the presence of 30‐fold molar excess of DEPC. After hydroxylamine treatment (which removes modifications from three His residues), two labels remain bound at the peptide N terminus and Lys16. DEPC treatment of Aβ1–42 promotes peptide aggregation, as monitored through the loss of soluble Aβ42 in a semi‐quantitative MALDI‐TOF MS assay. It has been previously proposed that Cu(II) ions protect Aβ1–16 from DEPC modification through binding to His6. We confirmed that Cu(II) ions decrease the stoichiometry of Aβ1–16 modification with the excess of DEPC being lower as compared to the control, which indicates that Cu(II) protects Aβ from DEPC modification. Sequencing of obtained Cu(II)‐protected Aβ1–16 samples showed that Cu(II) does not protect any residues completely, but partially protects all three His residues and the N terminus. Thus, the protection by Cu(II) ions is not related to specific metal binding to a particular residue (e.g. His6), but rather all His residues and the N terminus are involved in binding of Cu(II) ions. These results allow us to elucidate the effect of DEPC modification on amyloidogenity of human Aβ and to speculate about the role of His residues in these processes.

## Graphical abstract

Aβ1–42 can be modified with diethyl pyrocarbonate (DEPC) at three His residues, Lys16 and N terminus, and this treatment promotes peptide aggregation. Cu(II) ions protect Aβ1–16 from DEPC modification, and sequencing of obtained samples showed that Cu(II) did not protect any residues completely, but all three His and N terminus were partially protected.

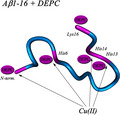

**Abbreviations**
AD: Alzheimer’s disease
Aβ: amyloid beta
DEPC: diethyl pyrocarbonate
ESI Q‐TOF MS/MS: electrospray ionization quadrupole time‐of‐flight tandem mass spectrometry
HEPES: 4‐(2‐hydroxyethyl)‐1‐piperazineethanesulfonic acid
HFIP: 1,1,1,3,3,3‐hexafluoro‐2‐propanol
MALDI‐TOF MS: matrix‐assisted laser desorption/ionization time‐of‐flight mass spectrometry
ThT: thioflavin T
α‐CHCA: α‐cyano‐4‐hydroxycinnamic acid

Alzheimer disease (AD) is the most common neurodegenerative disease, which is believed to start with the pathological build‐up of cerebral extracellular amyloid plaques, comprised of aggregated amyloid‐β peptides (Aβ) [1]. Enormous efforts have been directed to unravelling the factors initiating the formation of amyloid plaques, which is of crucial importance for understanding the pathological mechanisms of AD and for drug design. According to the accumulated knowledge, the path of protein/peptide self‐assembly is determined by a multitude of factors including the composition and sequence of its amino acid residues, concentration and by environmental conditions and interactions with external ligands such as metal ions. It is firmly established that metal ions such as Zn(II) and Cu(II) interact with Aβ peptides and initiate peptide aggregation mainly into nonfibrillar aggregates [2, 3]. These aggregates tend to fibrillize, and according to many authors, such pathway might initiate the formation of amyloid plaques also *in vivo* conditions [3, 4].

Structural and metal‐binding studies have demonstrated that the metal binding to Aβ peptides is at large extent determined by His residues located in positions 6, 13 and 14 of human Aβ [2, 3, 5, 6]. Replacement of His13 with Arg in rat Aβ peptide is assumed to be responsible for its lower sensitivity towards Zn(II)‐induced aggregation as compared with human Aβ [7]. It has also been shown that substoichiometric amounts of Cu(II) accelerate fibrillization [8] through binding to all three His residues, whereas His6 has been identified as a key ligand together with peptide N terminus [9]. Modification of these His residues can inhibit Cu(II)‐induced aggregation [10]. In addition, a large body of evidence indicates that binding of Cu(II) ions to Aβ peptides is not characterized by a single coordination mode but occurs through a population of multiple binding modes, which depends on solution pH and the nature of the residues in the N‐terminal region. In result, several distinct combinations of all three His residues, terminal Asp, N‐terminal amino and other groups can act as co‐ligands for Cu(II) [3, 11, 12, 13].

Chemical modification of amino acid residues is a classical protein chemistry approach for studies of the role of amino acid residues in the functioning of proteins/peptides, whereas several different reagents have been elaborated for all chemically reactive amino acid residues. Standard reagent for modification of His residues in proteins [14, 15, 16] is diethyl pyrocarbonate (DEPC), which results in the formation of *N*‐ethoxyformylimidazole [6, 14, 17] (Fig. 1).

**Fig. 1:**
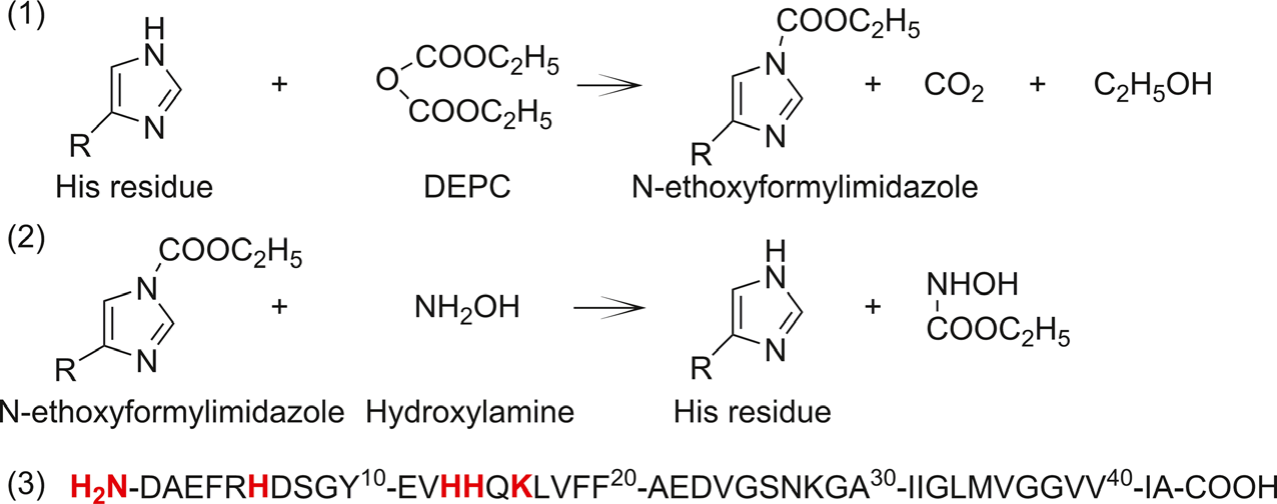
Chemical modification of His side chain with DEPC (1) and its removal by hydroxylamine (2) [27]. The sequence of human Aβ42 (3).

It should be noted that DEPC is not absolutely selective to His and can also modify other amino acid residues such as Lys, Tyr, Ser, Thr and also the N terminus at neutral pH [14]. Most of the DEPC modifications (except Lys and N terminus) are reversible and could be removed by nucleophilic agents such as hydroxylamine [18, 19, 20]. As a rule, DEPC can modify up to 30% of all amino acid residues in the average protein [21], and because of bulkiness, DEPC can react only with amino acid residues located on the surface of the protein [20]. DEPC has also been used for modification of amyloidogenic peptides [6, 17, 22, 23], which affects in case of insulin also peptide amyloidogenity [6]. Aβ has also been modified by DEPC, and it has been demonstrated that Cu(II) binding with the participation of His6 protects Aβ from modification [24]. However, the reaction stoichiometry has not been carefully analysed and it was not considered that DEPC can modify in Aβ at least four residues, involved in the binding of metal ions – three His and N terminus. Moreover, the effect of DEPC modification on amyloidogenity of Aβ has not been evaluated.

In the current study, we monitored the products of Aβ modification with DEPC in the presence and absence of Cu(II) ions by matrix‐assisted laser desorption/ionization time‐of‐flight mass spectrometry (MALDI‐TOF MS), determined the sites of modification by eelectrospray ionization quadrupole time‐of‐flight tandem MS (ESI Q‐TOF MS/MS) and performed comparative fibrillization studies of modified and native peptide. Obtained results allow to elucidate the effect of DEPC modification on inherent and metal‐induced amyloidogenity of human Aβ and to speculate about the role of His residues in these processes.

## Materials and methods

### Materials

Lyophilized Aβ1–42 and Aβ1–16 peptide were purchased from rPeptide (Watkinsville, GA, USA). Bovine insulin, DEPC; 1,1,1,3,3,3‐hexafluoro‐2‐propanol (HFIP); sodium chloride; sodium hydroxide; ammonium hydroxide; sodium phosphate monohydrate; disodium phosphate heptahydrate; α‐cyano‐4‐hydroxycinnamic acid (α‐CHCA); acetonitrile; trifluoroacetic acid; thioflavin T (ThT); and hydroxylamine hydrochloride (NH_2_OH·HCl) were purchased from Sigma‐Aldrich (St. Louis, MO, USA).

### Sample preparation

Lyophilized Aβ1–42 and Aβ1–16 were dissolved in HFIP at a concentration of 100 μm, divided into aliquots, HFIP was evaporated in vacuum, and the tubes with Aβ film were stored at −80 °C. HFIP treatment is used to disassemble peptide aggregates into monomers.

### Preparation of samples for MALDI‐TOF MS

HFIP‐treated Aβ1–42 and Aβ1–16 aliquots were dissolved in 10 mm NaOH, incubated for 10 min on ice and diluted to the final concentration of 10 µm with 50 mm phosphate buffer. DEPC was dissolved in ethanol and aliquoted in the argon‐filled glovebox to the required concentrations to avoid hydrolysis of DEPC by the moisture in the air. Aliquots were closed airtight in an argon atmosphere and stored at −20 °C. DEPC was added to the mixed sample (final concentration 20–300 µm) so that the volume of ethanol did not exceed 5% of total sample volume. Samples were incubated at 25 °C, and at distinct time points, 1 μL of the sample was mixed with 3 µL of α‐CHCA matrix (10 mg·mL^−1^) and analysed with MALDI‐TOF MS. In the case of Cu(II) protection experiments, three times molar excess of Cu(II) acetate was added to Aβ1–16 before the addition of DEPC.

Bovine insulin was weighed out and dissolved in 50 mm phosphate buffer to a final concentration of 10 µm.

To study the effect of hydroxylamine on DEPC modification, hydroxylamine (final concentration 0.5 m) was added to the samples, and at distinct time points (5–30 min), samples were analysed with MALDI‐TOF MS.

For semi‐quantitative MALDI‐TOF MS experiments, 1 μL was taken from the fluorescence spectroscopy samples, previously desalted with 1 mL PD SpinTrap G‐25 Column, and mixed with 3 µL of α‐CHCA matrix.

The MALDI‐TOF MS matrix α‐CHCA was dissolved in 60% acetonitrile containing 0.3% trifluoroacetic acid concentration 10 mg·mL^−1^. Hydroxylamine stock solution was prepared in ultrapure water at a concentration of 10 m.

### Preparation of samples for fluorescence spectroscopy

Aβ–4242 was dissolved in 10 mm NaOH, incubated 10 min on ice and diluted with 50 mm phosphate buffer, pH 7.4, to a final concentration of 40 μm and total volume of 140 μL. In the case of DEPC modification, DEPC was added (30 times excess), the sample was incubated for 30 s or 60 min, and the reaction was stopped with the addition of 10 mm imidazole. All samples (control, 30‐second DEPC‐modified and 60‐min DEPC‐modified Aβ1–42) were applied to 1 mL PD SpinTrap G‐25 Column (GE Healthcare, Chicago, IL, USA) equilibrated with 20 mm 4‐(2‐hydroxyethyl)‐1‐piperazineethanesulfonic acid (HEPES) buffer containing 100 mm NaCl, pH 7.3, and spun down for 1 min at 800 ***g***. The desalted fraction of Aβ1–42 (140 μL) was diluted with 20 mm HEPES buffer containing 100 mm NaCl, pH 7.3, to required volume of 500 μL.

### Preparation of samples for ESI Q‐TOF MS/MS sequencing

To locate DEPC modifications in Aβ by ESI Q‐TOF MS/MS sequencing, Aβ1–16 was used. Five different samples were prepared: (a) Aβ1–16 control, (b) DEPC‐modified Aβ1–16, (c) DEPC‐modified and hydroxylamine‐treated Aβ1–16, (d) copper protected DEPC‐modified Aβ1–16 and (e5) copper‐protected, DEPC‐modified and hydroxylamine‐treated Aβ1–16.

DEPC‐modified samples were prepared as follows: Aβ1–16 film was dissolved in 10 mm NaOH, incubated 10 min on ice, diluted with 50 mm phosphate buffer, pH 7.4, to a final concentration of 40 μm and DEPC was added in 30 times molar excess. DEPC modification was carried out for 1 hour, and reaction products were monitored with MALDI‐TOF MS. This procedure resulted in Aβ1–16 modified with DEPC in 4 and 5 positions. This sample was treated with hydroxylamine (final concentration 0.5 m m) to remove DEPC modifications from His residues. Hydroxylamine treatment was carried out for 30 min and monitored with MALDI‐TOF MS. This resulted in Aβ1–16 modified by DEPC in 1 and 2 positions.

Cu(II)‐protected DEPC‐modified Aβ1–16 was prepared as follows: Aβ1–16 was dissolved in 10 mm NaOH, incubated 10 min on ice, diluted with 50 mm phosphate buffer, pH 7.4, to a final concentration of 40 μm, 120 μm copper(II)acetate was added and the sample was incubated for 10 min. DEPC modification with 30 times molar excess was then carried out for 1 hour, and the reaction was monitored with MALDI‐TOF MS. This procedure resulted in Aβ1–16 modified with DEPC in 2 and 3 positions. The resulting sample was further treated with hydroxylamine for 30 min and analysed with MALDI‐TOF MS.

Before ESI Q‐TOF MS/MS experiments, all samples were desalted with 1 mL PD SpinTrap G‐25 Column (GE Healthcare), equilibrated with 20 mm ammonium acetate, pH 7.4. Resulting samples were diluted 3 times with 20 mm ammonium acetate, pH 7.4.

### Monitoring of peptide fibrillization by fluorescence spectroscopy

Fibrillization of Aβ1–42 and DEPC‐modified Aβ1–42 was studied by using fluorescent ligand ThT, which fluorescence intensity at 480 nm (excitation at 440 nm) is increased upon binding to amyloid fibrils. ThT fluorescence was monitored on a Perkin Elmer (Waltham, MA, USA) LS45 fluorescence spectrophotometer in 500‐µL cuvette by constant stirring at 40 °C. To study the effect of DEPC modification on Aβ1–42 fibrillization, three samples were compared: desalted Aβ1–42 control, desalted 30‐s DEPC‐modified Aβ1–42 and desalted 1‐hour DEPC‐modified Aβ1–42.

### MALDI‐TOF MS

The reaction mixtures were analysed by MALDI‐TOF MS on Bruker Autoflex and Microflex LT instruments (Bruker Corporation, Billerica, MA, USA). One microliter of the reaction mixture was mixed with 3 μL of α‐CHCA matrix, and 1 μL of the mixture was pipetted to the MALDI‐TOF MS plate and dried in air. In semi‐quantitative experiments, α‐CHCA matrix contained 0.3 μm bovine insulin as an internal standard. Parameters are as follows: *m*/*z* range from 100 to 10 000 in linear positive mode, 337‐nm laser frequency 60 Hz, ions source voltages 10 and 9.1 kV, lens voltage 3 kV and 1000 shots accumulated per spectrum.

### ESI Q‐TOF MS/MS

Samples of Aβ1–16 and DEPC‐modified Aβ1–16 in 20 mm ammonium acetate buffer, pH 7.4, were injected into the electrospray ion source of an Agilent Technology 6540 UHD Accurate‐Mass Q‐TOF MS instrument by a syringe pump at 7 μL·min^−1^. The spectrometer parameters were as follows: drying gas temperature 100 °C, drying gas 4 L·min^−1^, nebulizer 15 psig, skimmer voltage 65 V, capillary voltage 3500 V and fragmentor 400 V (capillary 3228 uA, Okt 1 RF Vpp 750 V). ESI MS spectra were recorded for 10 min in the region of 500–3000 *m*/*z*, and fragmentation spectra were recorded in 50–3000 *m*/*z* region. The peptides were fragmented with collision energy (CID) of 50 in case of DEPC‐modified samples and 45 for the control sample. Obtained fragmentation spectra peaks were analysed by mmass open‐source MS tool [25] using 1% of the largest peaks from ESI Q‐TOF MS/MS spectra with fragment matching error tolerance 0.01 Da.

## Results and Discussion

First, the conditions for the modification reaction were optimized, which was necessary for the determination of the maximal stoichiometry of the DEPC reaction and synthesis of peptides with defined modification pattern for fibrillization studies. In an earlier study, a two‐fold excess of DEPC in phosphate buffer at pH 6.8 was used for peptide modification [6]; however, in these conditions, the yield of modification is very low (Fig. S1A). The modification was substantially more efficient at pH 7.4 (See Fig. S1B), and therefore, the latter pH was selected for our studies.

**Fig. 2:**
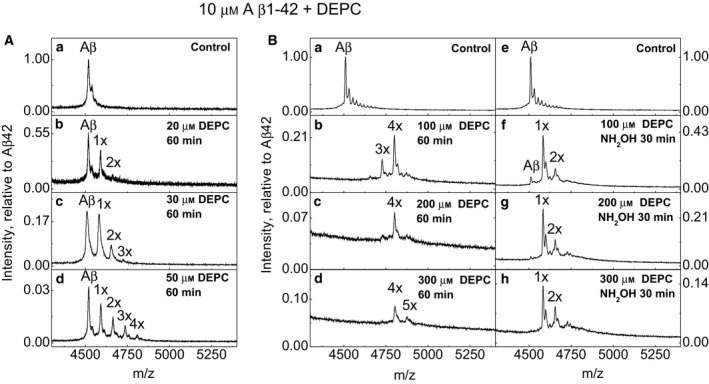
MALDI‐TOF MS spectra of Aβ1–42 modified by increasing concentrations of DEPC in phosphate buffer at pH 7.4: molar excess of two times (2×) (A, b), 3× (A, c), 5× (A, d), 10× (B, b), 20× (B, c) and 30× (B, d) and hydroxylamine treatment of 10×, 20× and 30× DEPC samples, respectively (B, f, g, h). Annotations 1–5× denote the number of DEPC modifications bound to Aβ. Intensity is relative to Aβ42 in the control sample.

Results presented in Fig. 2 demonstrate that the stoichiometry of DEPC modification depends substantially from the concentration of DEPC – after 1‐hour incubation with two times molar excess of DEPC, the main peak in the spectrum still corresponded to the unmodified Aβ (Fig. 2A,B), whereas modification of the peptide at four and five positions was achieved in the presence of 20‐ to 30‐fold molar excess of DEPC (Fig. 2B,C,D). This result indicates that in addition to three His residues, maximums of two additional amino acid residues are modified in Aβ1–42 by DEPC.

It is known that hydroxylamine removes the carbethoxy group from all amino acid residues except Lys and peptide N terminus [19]. After hydroxylamine treatment, one and two modifications remained in case of Aβ1–42 carrying four and five DEPC modifications, respectively (Fig. 2,B,G,H). Most probably these two modifications occur at N‐terminal amino group and the amino group of Lys16.

**Fig. 3:**
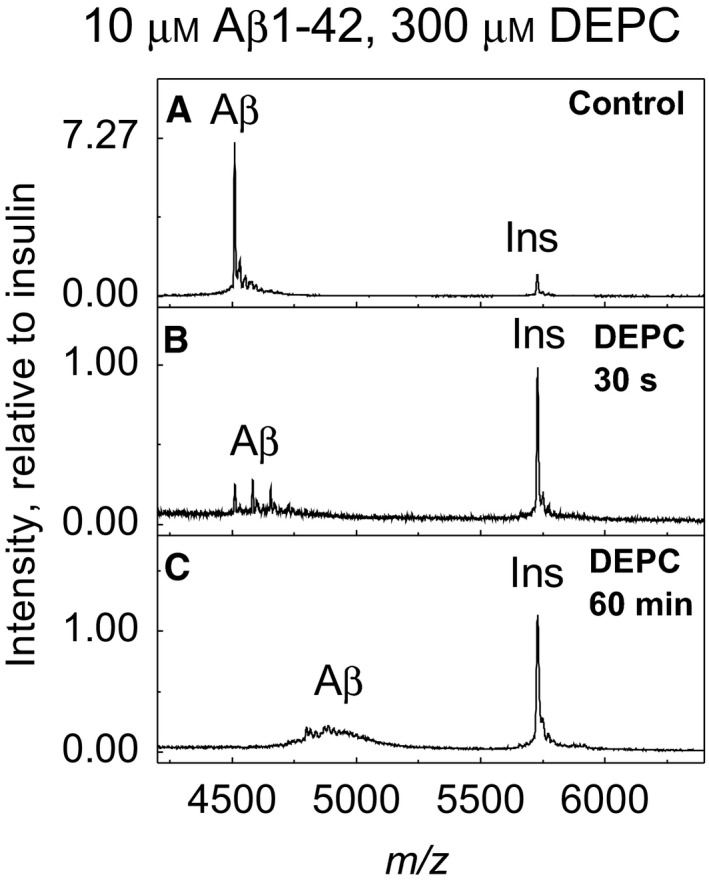
Semi‐quantitative MALDI‐TOF MS assay for peptide aggregation. Desalted Aβ1–42 as control (A), desalted Aβ1–42 modified with 30× DEPC for 30 s (B) and desalted Aβ1–42 modified with 30× DEPC for 60 min (C). Intensity is relative to insulin in the control sample.

We also observed that treatment of Aβ1–42 with 20‐ and 30‐fold molar excess of DEPC leads to the decrease in MALDI‐TOF MS signal (Fig. 2,B,C,D), which might be caused by peptide aggregation since the fibrils do not show peptide peaks in MALDI‐TOF MS under ordinary conditions [26]. A semi‐quantitative MALDI‐TOF MS experiment by using of 0.3 μm insulin as an internal standard demonstrated that indeed, the decrease in signal intensity of DEPC‐modified Aβ1–42 is connected with the decrease in the concentration of soluble Aβ1–42 (Fig. 3).

**Fig. 4:**
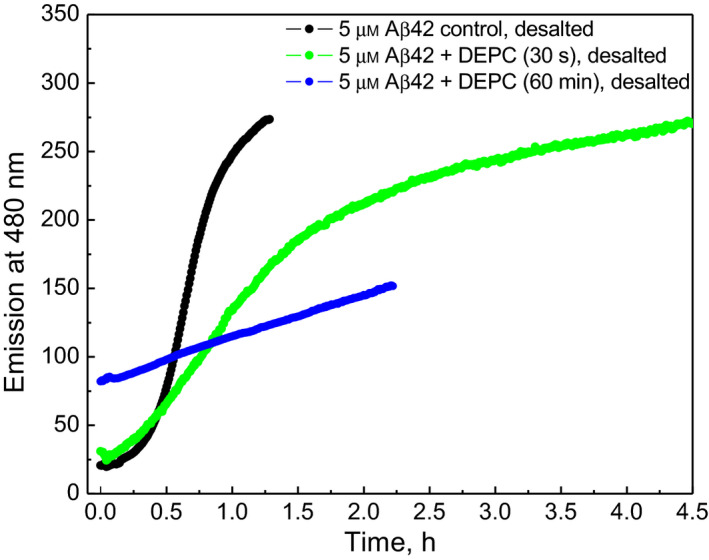
The fibrillization of DEPC‐modified Aβ1–42 in agitated conditions. Desalted Aβ1–42 control (black line), and Aβ1–42 incubated for 30 s (green line) and 60 min (blue line) with 30× excess of DEPC. Conditions: 20 mm ammonium acetate, pH 7.4, temperature 40 °C, constant stirring.

Fibrillization potential of Aβ peptides was also studied in agitated conditions by using ThT fluorescence assay. Fibrillization of normal Aβ1–42 (Fig. 4 black line) was compared to DEPC‐modified Aβ1–42 and incubated for 30 s (Fig. 4 green line) and 60 min (Fig. 4 blue line) with 30× excess of DEPC. Aβ1–42 modified with DEPC for 30 s, which represents partially modified Aβ1–42, showed similar lag time but a two‐fold slower elongation rate compared with the control sample. Aβ1–42 modified with DEPC for 60 min, representing fully modified Aβ1–42, exposes an initial increased fluorescence and even slower elongation rate. The initial fluorescence is indicative for the presence of fibrils in the sample and decreased fibrillization rate shows that higher modification level decreases the fibrillization rate under agitated conditions. Results indicate that DEPC modification decreases the fibrillization rate of Aβ1–42 in agitated conditions; however, it promotes peptide aggregation.

**Fig. 5:**
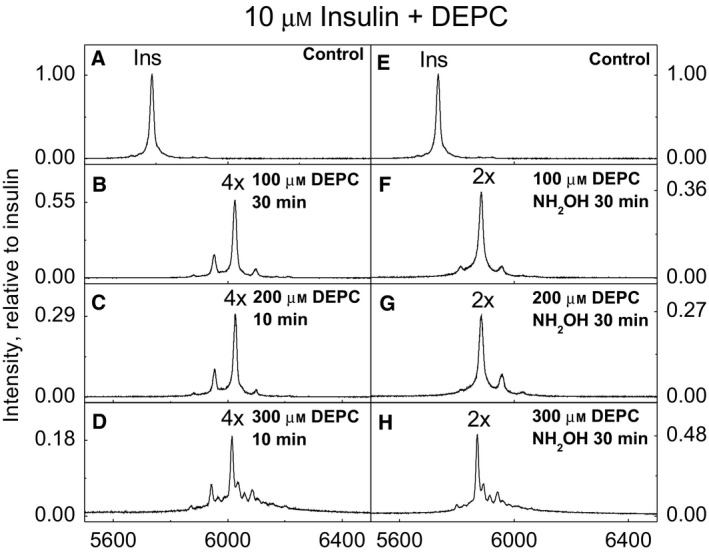
MALDI‐TOF MS spectra of bovine insulin modified with DEPC in phosphate buffer at pH 7.4: DEPC excess of 10× (B), 20× (C) and 30× (D) and hydroxylamine treatment of 10×, 20× and 30× DEPC‐modified insulin samples, respectively (F, G, H), compared with insulin control (A, E). Annotations 1–5× denote the number of DEPC modifications added to insulin. Intensity is relative to insulin in the control sample.

DEPC modification of insulin was faster as compared to Aβ1–42: in case of 10‐fold molar excess of DEPC (Fig. 5B), the insulin modification was completed in 30 min and in case of 20‐ and 30‐fold molar excess of DEPC (Fig. 5C,D), it was completed in 10 min. The main peak in all experiments was insulin modified with four DEPC molecules, and after hydroxylamine treatment, two modifications remained (Fig. 5F–H).

### Sequencing of DEPC‐modified Aβ with ESI Q‐TOF MS/MS

**Fig. 6:**
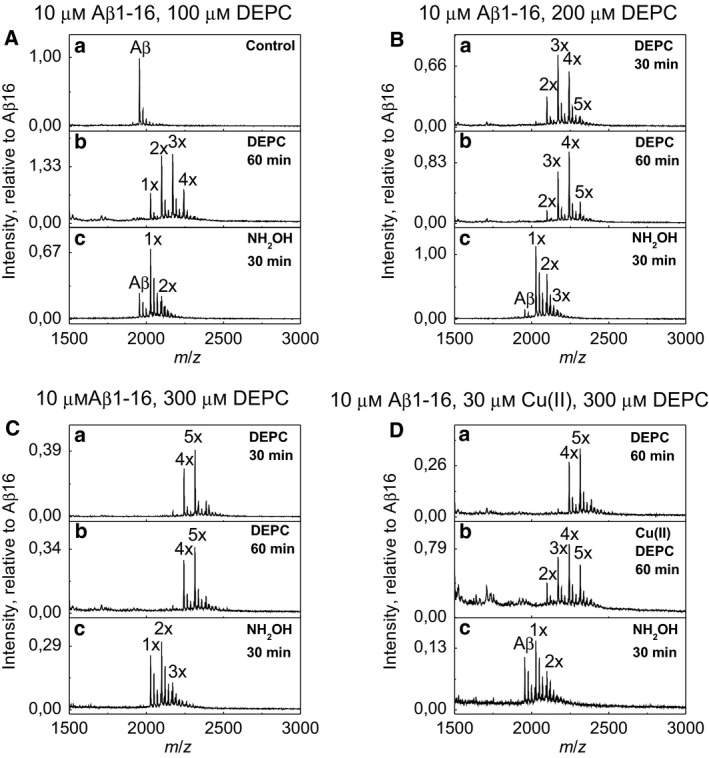
MALDI‐TOF MS spectra of Aβ1–16 DEPC modification in phosphate buffer pH 7.4 with different DEPC concentrations: excess of 10× (A), 20× (B) and 30× (C) and Cu(II) protection of 30× DEPC (D). Annotations 1–5× denote the number of DEPC modifications added to Aβ. Intensity is relative to Aβ16 in the control sample.

In order to locate the DEPC modification sites in Aβ peptide, shorter peptide Aβ1–16 was studied because of its better solubility and a lower tendency for aggregation. 10‐fold (Fig. 6A), 20‐fold (Fig. 6B) and 30‐fold (Fig. 6C) molar excess of DEPC was studied by MALDI‐TOF MS, showing the maximum level of modification to be reached in case of 30‐fold molar excess of DEPC. We used ESI Q‐TOF MS/MS and sequenced Aβ1–16 control (Fig. S2), the major Aβ1–16 form modified with four DEPC molecules and a minor form modified with five DEPC molecules (Fig. 6C,B). We also sequenced the Aβ1–16 with a single modification obtained from the major form after hydroxylamine treatment (Fig. 6,C,C). Sequencing results obtained by mmass indicate that in Aβ1–16 form carrying five DEPC modifications, three His residues, Lys16 and N terminus were modified (Fig. S5). In Aβ1–16 with four modifications, only two His residues (His6 and His14), N terminus and Lys were modified (Fig. S4). Sequencing of Aβ1–16 sample carrying one modification after hydroxylamine treatment indicated that only the N terminus was modified (Fig. S3) compared with Aβ1–16 control (Fig. S2). This result indicates that the N terminus of Aβ1–16 is modified faster than Lys16.

Our results showed that Cu(II) ions decrease the stoichiometry of Aβ1–16 modification with an excess of DEPC (Fig. 6,D,A,B) in average by two units, showing that Cu(II) protects Aβ residue(s) from DEPC modification. Sequencing of obtained Aβ1–16 samples containing two and three modifications showed that there is a mixture of Aβ1–16 species with a different distribution of DEPC modifications, which indicates that Cu(II) does not protect any residues completely but it protected partially all three His residues and N terminus (Figs S6 and  S7). Thus, the protection by Cu(II) ions indicates that all His residues, as well as N terminus of Aβ1–16, are involved in binding of Cu(II) ions. This is expected as Cu(II) ions prefer coordination with 4–6 groups, and moreover, Aβ peptides do apparently not contain a specific binding site for metal ions like it exists in enzymes, but is assumingly binding Cu(II) ions into multiple coordination environments, which are energetically similar and in equilibrium [3, 11, 12, 13]. In earlier work, the Cu(II) protection on DEPC modification of Aβ1–16 peptide was assumed to occur with the participation of His6 only [24]. However, in that work, only the peptide derivative with a single DEPC modification (mainly at His6) was used in Cu(II) protection experiment. Sequencing of corresponding peptides showed that His6 is protected by Cu(II); however, these data cannot show the protection of His13, His14 and N terminus since these residues were not modified in the absence of Cu(II) ions. Thus, our experiments with high‐level DEPC‐modified samples indicate that the protection by Cu(II) ions is not connected with the involvement of only His6, but all His residues and N terminus are involved.

ESI Q‐TOF MS/MS sequencing data in Figs S3 and S8 show that after hydroxylamine treatment of DEPC‐modified Aβ1–16, only N terminus remained modified, whereas in the presence of Cu(II) a mixture of N terminus and Lys16‐modified Aβ1–16 was detected.

## Conclusions

MALDI‐TOF MS results show that Aβ1–42 could be modified with maximum five DEPC molecules, and our ESI Q‐TOF MS/MS sequencing identified that three His residues, Lys16 and N terminus of Aβ are modified. Aβ1–42 with four DEPC modifications had only two His residues (His6 and His14) modified in addition to Lys16 and N terminus. Hydroxylamine treatment removed only modifications from His residues.

DEPC treatment of Aβ1–42 promotes peptide aggregation monitored through the loss of soluble Aβ1–42 in a semi‐quantitative MALDI‐TOF MS assay; however, according to ThT assay, the DEPC modification decreased the rate of Aβ1–42 fibrillization.

Cu(II) ions decrease the stoichiometry of Aβ1–16 modification with an excess of DEPC by average 2 units, confirming that Cu(II) protects Aβ from DEPC modification. Sequencing of Cu(II)‐protected Aβ1–16 revealed that all three His residues and the N terminus of Aβ1–16 were partially protected, which indicates their involvement in the binding of Cu(II) ions.

## Conflict of interest

The authors declare no conflict of interest.

## Author contributions

VT and PP conceived and designed the project; MF acquired the data; MF, VT and PP analysed and interpreted the data; and MF, VT and PP wrote the paper.

## Supporting information

Since the MALDI‐TOF MS analysis of the products of DEPC modification of insulin under the conditions reported in an earlier study (2‐fold molar excess of DEPC, phosphate buffer pH 6.8) the reaction (Fig. S1A) showed that only a small amount of the peptide was modified by 1–3 DEPC molecules. Increasing the pH value from 6.8 to 7.4 increased the modification level substantially (Fig. S1B).

**Fig. S1.** MALDI‐TOF MS spectra of bovine insulin modified with a two‐times molar excess of DEPC in phosphate buffer at pH 6.8 (A) and pH 7.4 (B). Annotations 1–4× denote the number of DEPC modifications added to insulin. Intensity is relative to insulin in the control sample.

**Fig. S2.** Sequencing of Aβ1–16 control. Above is a spectrum of identified fragments from mmass and below is the table of mmass results from ESI Q‐TOF MS/MS spectrum where pink cells are false‐positive results indicated by mmass as DEPC modifications in Aβ1–16 control sample, grey cells are results found by mmass software for Aβ1–16 control. ‘0 DEPC’ row indicates peptide fragments without modifications and ‘1–5 DEPC’ indicates the number of modifications found by mmass software. The sample was in 20 mm ammonium acetate, pH 7.4; collision energy 45; auto sequencing.

**Fig. S3.** Targeted sequencing of Aβ1–16 modified with one DEPC molecule after hydroxylamine treatment. Above is a spectrum of identified fragments from mmass and below is the table of mmass results from ESI Q‐TOF MS/MS spectrum where pink cells are false‐positive results indicated by mmass as DEPC modifications in Aβ1–16 control sample, grey cells are results found by mmass software for Aβ1–16 modified with 1 DEPC molecule. ‘0 DEPC’ row indicates peptide fragments without modifications and ‘1–5 DEPC’ indicates the number of modifications found by mmass software. The sample was in 20 mm ammonium acetate, pH 7.4; precursor was peak 1014 *m*/*z* (once DEPC‐modified Aβ1–16 with charge 2+), collision energy 50.

**Fig. S4.** Targeted sequencing data from the peak of Aβ1–16 modified with 4 DEPC molecules. Above are spectra of identified fragments from mmass and below are tables of mmass results from ESI Q‐TOF MS/MS spectrum where pink cells are false‐positive results indicated by mmass as DEPC modifications in Aβ1–16 control sample, grey cells are results found by mmass software for Aβ1–16 modified with four DEPC molecules. ‘0 DEPC’ row indicates peptide fragments without modifications and ‘1–5 DEPC’ indicates the number of modifications found by mmass software. Samples were in 20 mm ammonium acetate, pH 7.4; precursor was peak 1122.5 *m*/*z* (four times DEPC‐modified Aβ1–16 with charge 2+), collision energy 50.

**Fig. S5.** Targeted sequencing data from the peak of Aβ1–16 modified with 5 DEPC molecules. Above are spectra of identified fragments from mmass and below are tables of mmass results from ESI Q‐TOF MS/MS spectrum where pink cells are false‐positive results indicated by mmass as DEPC modifications in Aβ1–16 control sample, grey cells are results found by mmass software for Aβ1–16 modified with five DEPC molecules. ‘0 DEPC’ row indicates peptide fragments without modifications and ‘1–5 DEPC’ indicates the number of modifications found by mmass software. Samples were in 20 mm ammonium acetate, pH 7.4; precursor was peak 1158.5 *m*/*z* (five times DEPC‐modified Aβ1–16 with charge 2+), collision energy 50.

**Fig. S6.** Targeted sequencing data from the peak of copper protected Aβ1–16 modified with two DEPC molecules. Above are spectra of identified fragments from mmass and below are tables of mmass results from ESI Q‐TOF MS/MS spectrum, where pink cells are false‐positive results indicated by mmass as DEPC modifications in Aβ1–16 control sample combined with results indicating higher modification level than the precursor. Grey cells are results found by mmass software for copper protected Aβ1–16 modified with two DEPC molecules. ‘0 DEPC’ row indicates peptide fragments without modifications and ‘1–5 DEPC’ indicates the number of modifications found by mmass software. Samples were in 20 mm ammonium acetate, pH 7.4; precursor was peak 1050.5 *m*/*z* (two times DEPC‐modified Aβ1–16 with charge 2+), collision energy 50.

**Fig. S7.** Targeted sequencing data from the peak of copper protected Aβ1–16 modified with three DEPC molecules. Above are spectra of identified fragments from mmass and below are tables of mmass results from ESI Q‐TOF MS/MS spectrum, where pink cells are false‐positive results indicated by mmass as DEPC modifications in Aβ1–16 control sample combined with results indicating higher modification level than the precursor. Grey cells are results found by mmass software for copper protected Aβ1–16 modified with three DEPC molecules. ‘0 DEPC’ row indicates peptide fragments without modifications and ‘1–5 DEPC’ indicates the number of modifications found by mmass software. Samples were in 20 mm ammonium acetate, pH 7.4; precursor was peak 1086.5 *m*/*z* (three times DEPC‐modified Aβ1–16 with charge 2+), collision energy 50.

**Fig. S8.** Targeted sequencing data from the peak of copper protected Aβ1–16 modified with 1 DEPC molecules after hydroxylamine treatment. Above is a spectrum of identified fragments from mmass and below is the table of mmass results from ESI Q‐TOF MS/MS spectrum, where pink cells are false‐positive results indicated by mmass as DEPC modifications in Aβ1–16 control sample combined with results indicating higher modification level than the precursor. Grey cells are results found by mmass software for copper protected Aβ1–16 modified with 1 DEPC molecule. ‘0 DEPC’ row indicates peptide fragments without modifications and ‘1–5 DEPC’ indicates the number of modifications found by mmass software. The sample was in 20 mm ammonium acetate, pH 7.4; precursor was peak 1014 *m*/*z* (one‐time DEPC‐modified Aβ1–16 with charge 2+), collision energy 50.
