# Supplementary figures and images for: Copper(II) partially protects three histidine residues and the N‐terminus of amyloid‐β peptide from diethyl pyrocarbonate (DEPC) modification
Source: FEBS Open Bio. 2020 Apr 29;10(6):1072–81. doi: 10.1002/2211-5463.12857 (PMC7262909; doi:10.1002/2211-5463.12857)

10  $\mu$ M Insulin, 20  $\mu$ M DEPC, pH=6.8

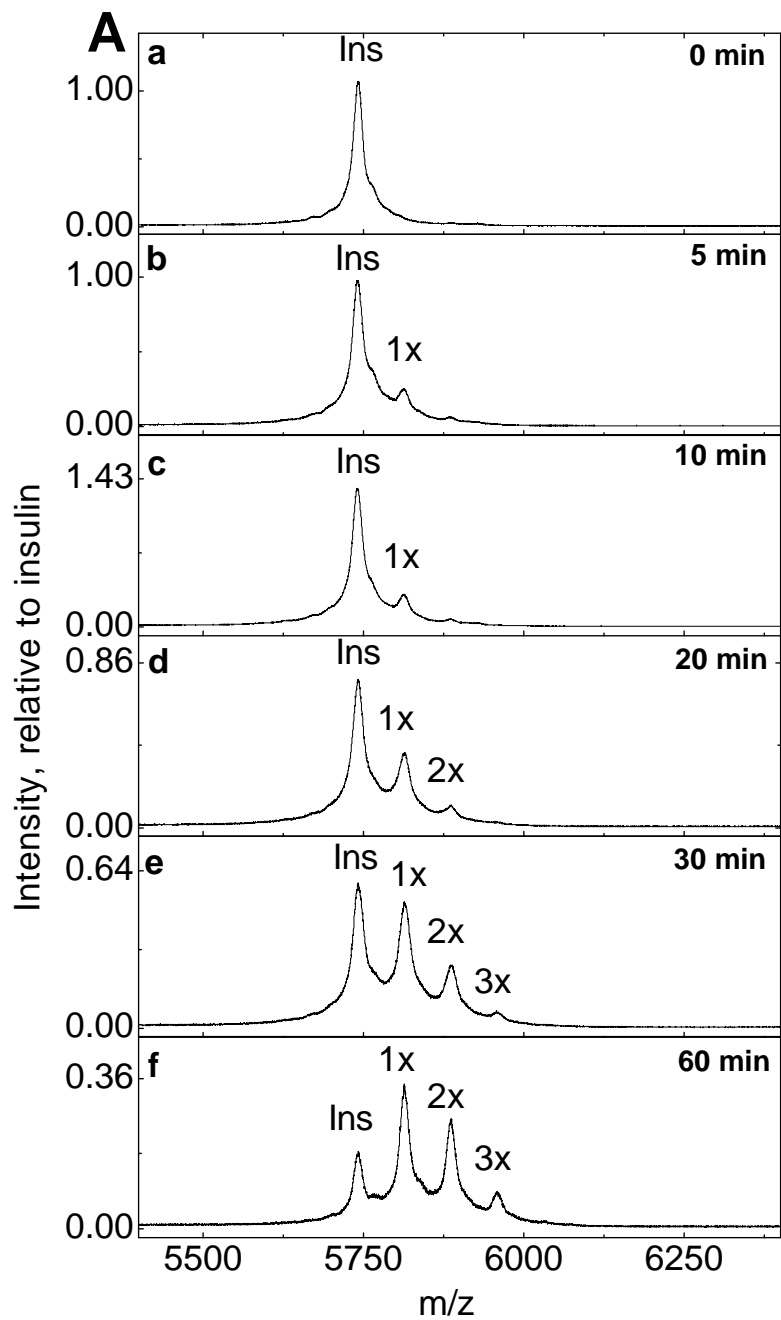

10  $\mu$ M Insulin, 20  $\mu$ M DEPC, pH=7.4

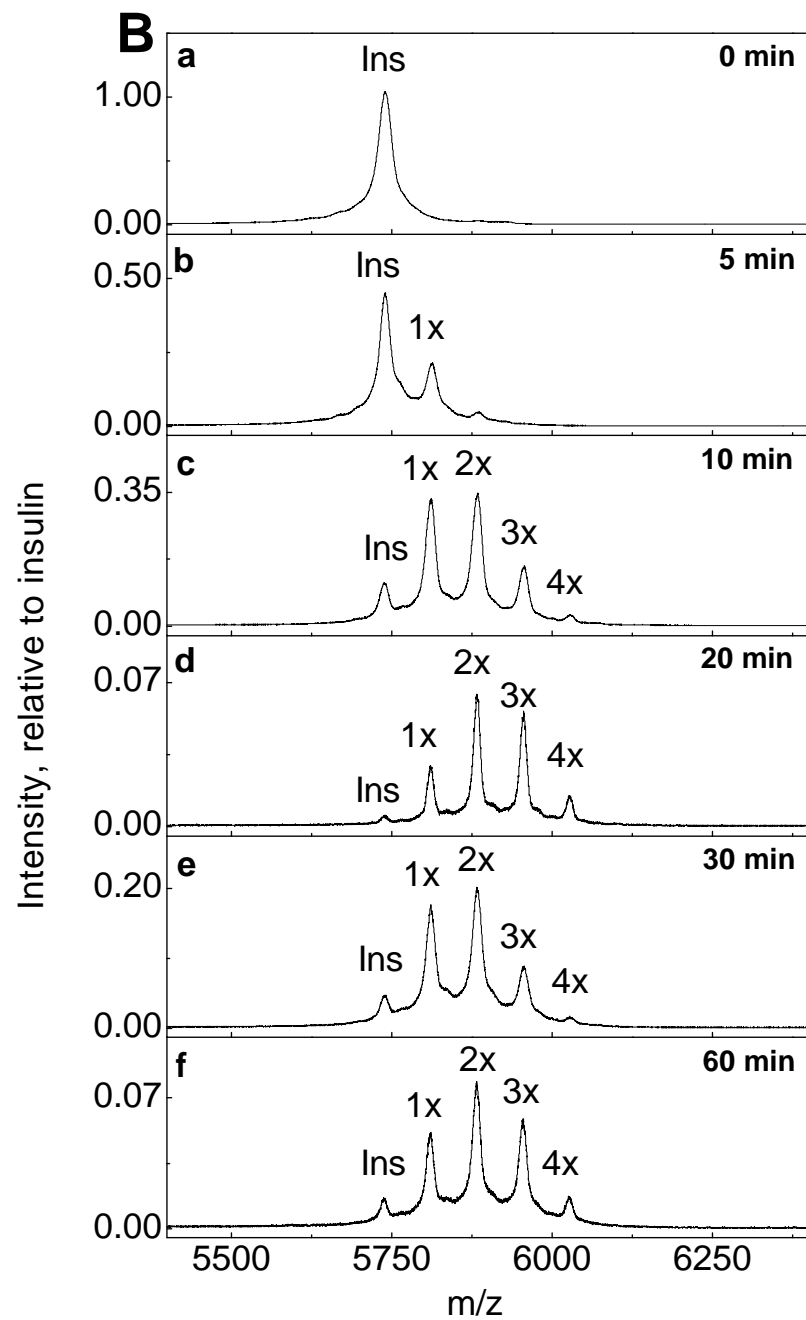

Supplement: Supplementary file 1 — Fig. S1. MALDI‐TOF MS spectra of bovine insulin modified with a two‐times molar excess of DEPC in phosphate buffer at pH 6.8 (A) and pH 7.4 (B). Annotations 1–4× denote the number of DEPC modifications added to insulin. Intensity is relative to insulin in the control sample. [file FEB4-10-1072-s001.pdf]

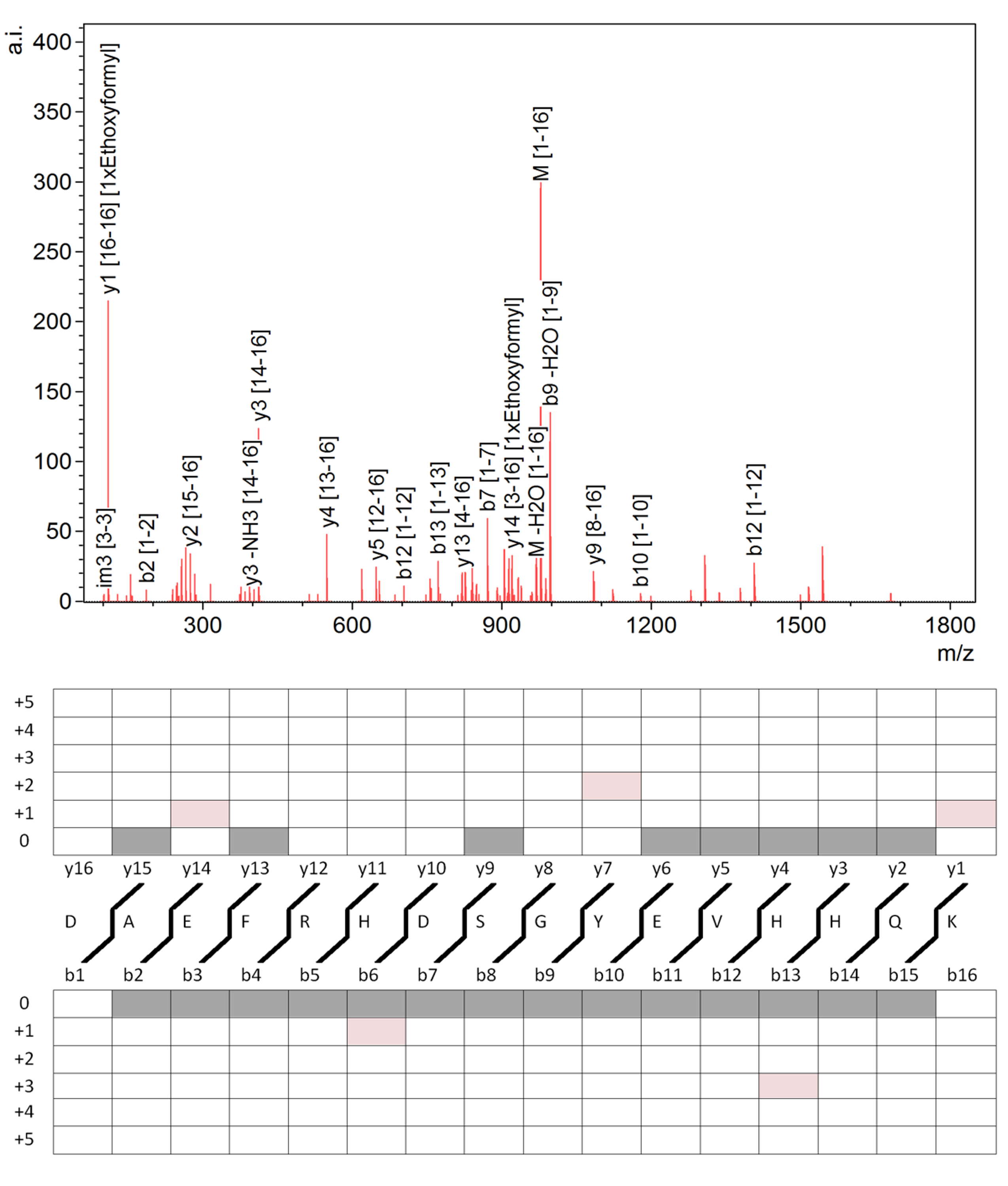

Supplement: Supplementary file 2 — Fig. S2. Sequencing of Aβ1–16 control. Above is a spectrum of identified fragments from mmass and below is the table of mmass results from ESI Q‐TOF MS/MS spectrum where pink cells are false‐positive results indicated by mmass as DEPC modifications in Aβ1–16 control sample, grey cells are results found by mmass software for Aβ1–16 control. ‘0 DEPC’ row indicates peptide fragments without modifications and ‘1–5 DEPC’ indicates the number of modifications found by mmass software. The sample was in 20 mm ammonium acetate, pH 7.4; collision energy 45; auto sequencing. [file FEB4-10-1072-s002.tif]

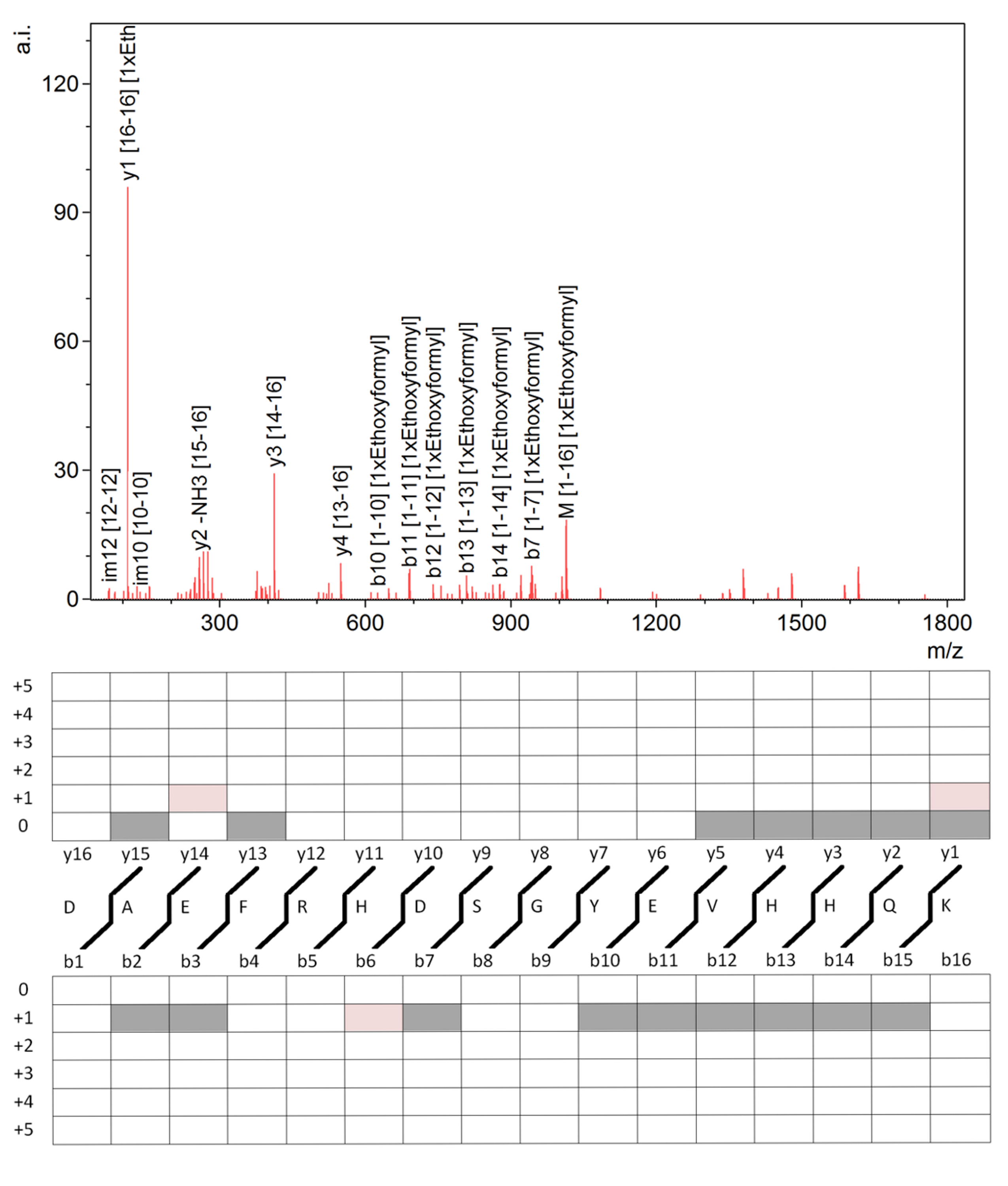

Supplement: Supplementary file 3 — Fig. S3. Targeted sequencing of Aβ1–16 modified with one DEPC molecule after hydroxylamine treatment. Above is a spectrum of identified fragments from mmass and below is the table of mmass results from ESI Q‐TOF MS/MS spectrum where pink cells are false‐positive results indicated by mmass as DEPC modifications in Aβ1–16 control sample, grey cells are results found by mmass software for Aβ1–16 modified with 1 DEPC molecule. ‘0 DEPC’ row indicates peptide fragments without modifications and ‘1–5 DEPC’ indicates the number of modifications found by mmass software. The sample was in 20 mm ammonium acetate, pH 7.4; precursor was peak 1014 m/z (once DEPC‐modified Aβ1–16 with charge 2+), collision energy 50. [file FEB4-10-1072-s003.tif]

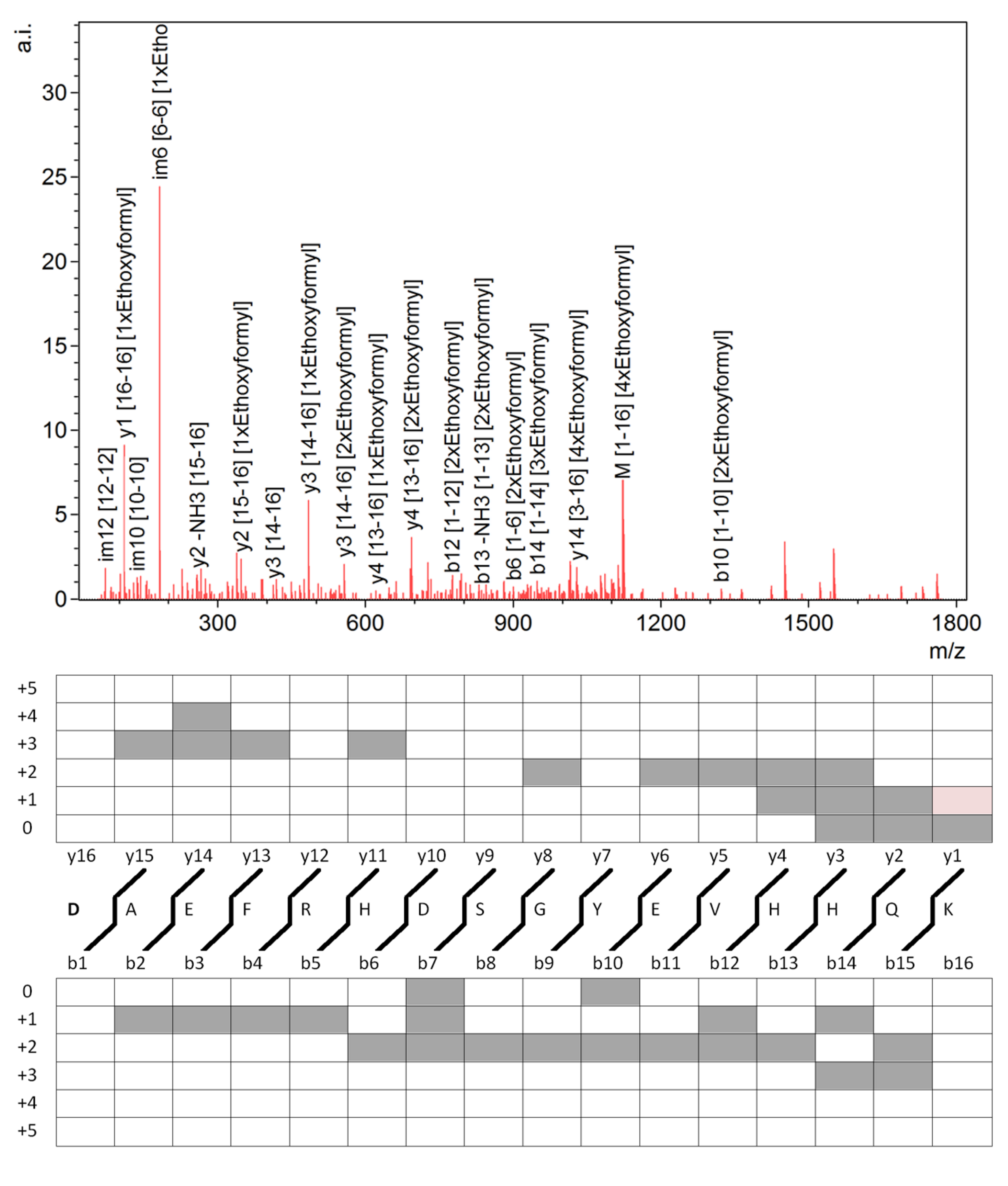

Supplement: Supplementary file 4 — Fig. S4. Targeted sequencing data from the peak of Aβ1–16 modified with 4 DEPC molecules. Above are spectra of identified fragments from mmass and below are tables of mmass results from ESI Q‐TOF MS/MS spectrum where pink cells are false‐positive results indicated by mmass as DEPC modifications in Aβ1–16 control sample, grey cells are results found by mmass software for Aβ1–16 modified with four DEPC molecules. ‘0 DEPC’ row indicates peptide fragments without modifications and ‘1–5 DEPC’ indicates the number of modifications found by mmass software. Samples were in 20 mm ammonium acetate, pH 7.4; precursor was peak 1122.5 m/z (four times DEPC‐modified Aβ1–16 with charge 2+), collision energy 50. [file FEB4-10-1072-s004.tif]

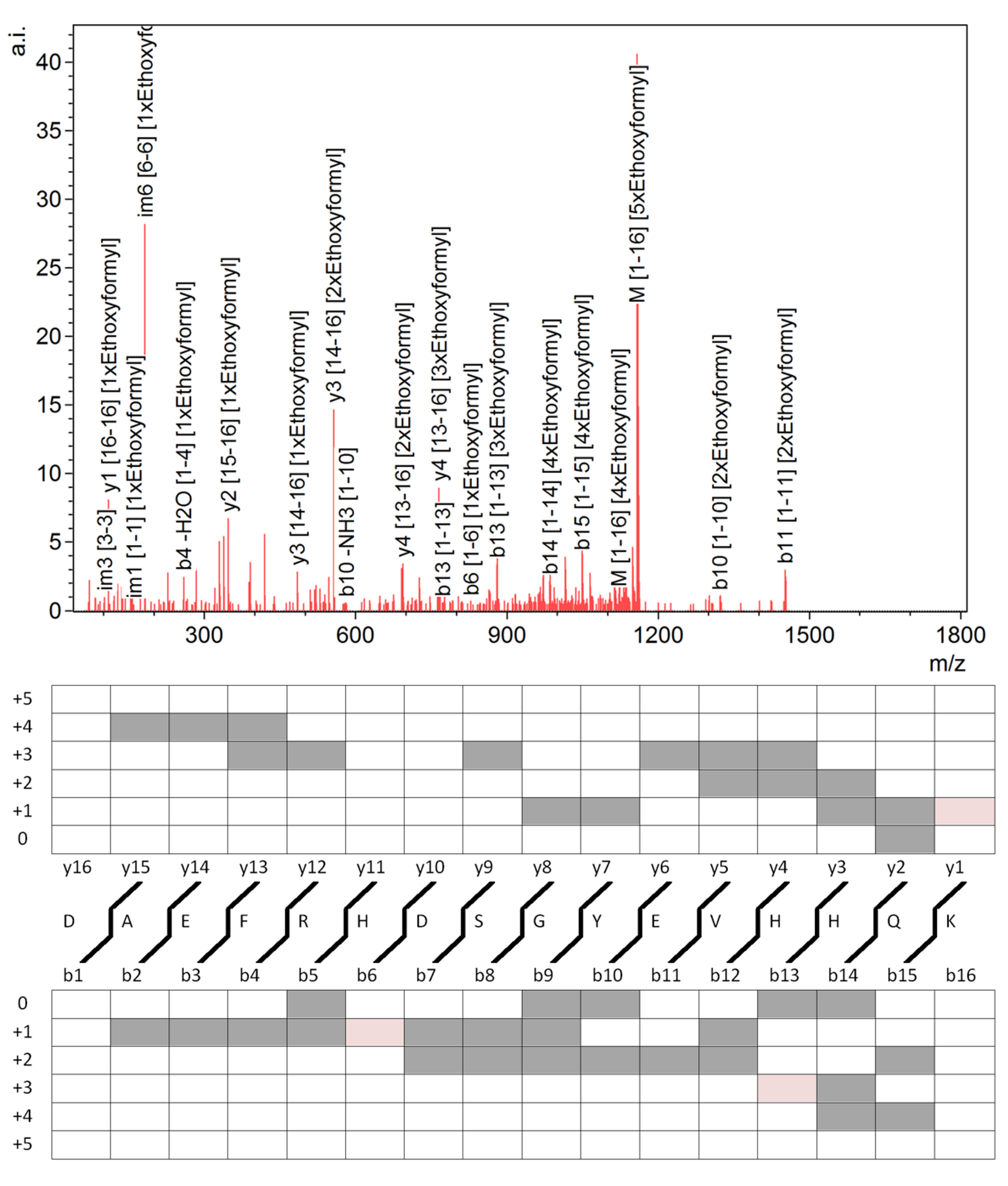

Supplement: Supplementary file 5 — Fig. S5. Targeted sequencing data from the peak of Aβ1–16 modified with 5 DEPC molecules. Above are spectra of identified fragments from mmass and below are tables of mmass results from ESI Q‐TOF MS/MS spectrum where pink cells are false‐positive results indicated by mmass as DEPC modifications in Aβ1–16 control sample, grey cells are results found by mmass software for Aβ1–16 modified with five DEPC molecules. ‘0 DEPC’ row indicates peptide fragments without modifications and ‘1–5 DEPC’ indicates the number of modifications found by mmass software. Samples were in 20 mm ammonium acetate, pH 7.4; precursor was peak 1158.5 m/z (five times DEPC‐modified Aβ1–16 with charge 2+), collision energy 50. [file FEB4-10-1072-s005.tif]

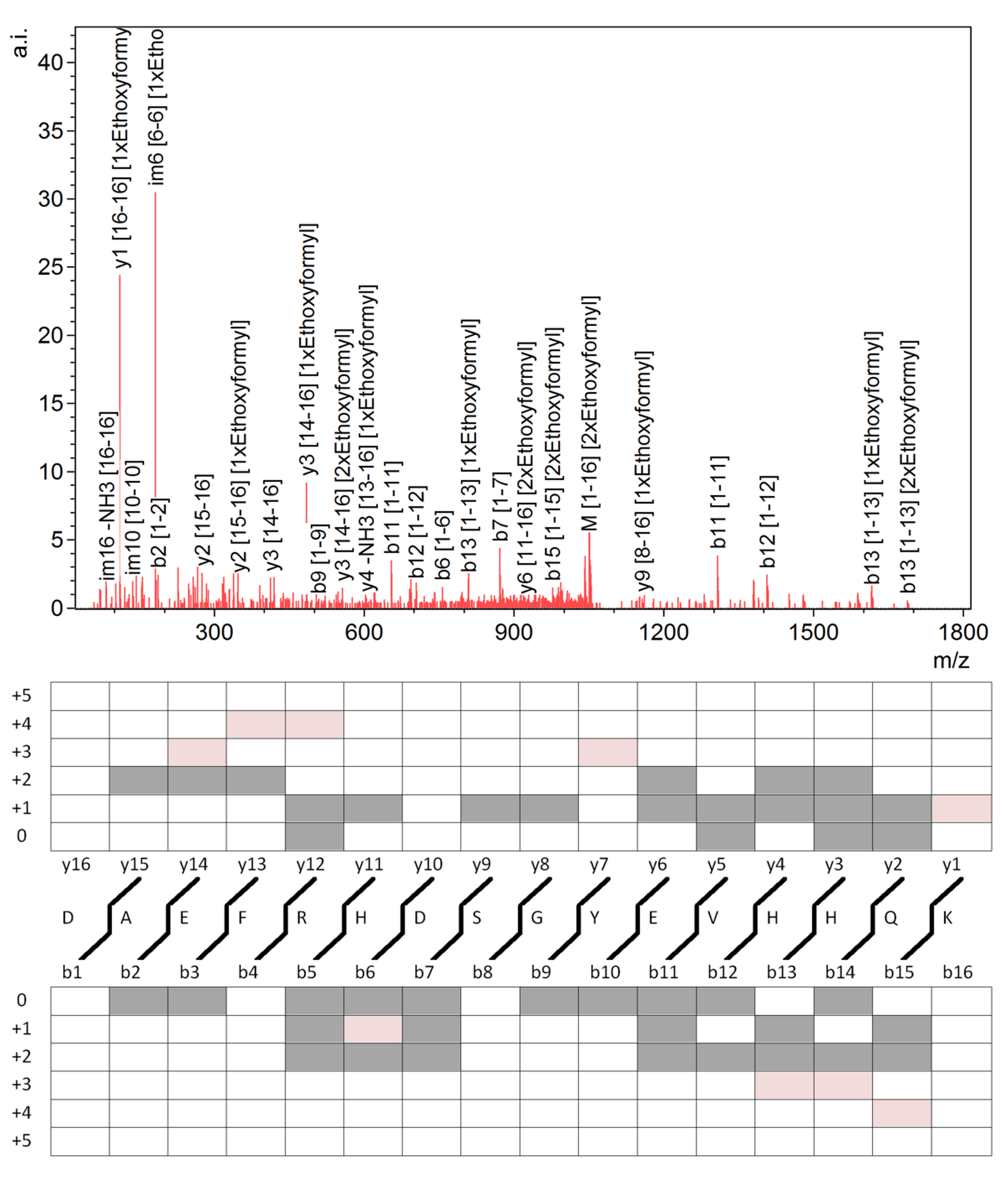

Supplement: Supplementary file 6 — Fig. S6. Targeted sequencing data from the peak of copper protected Aβ1–16 modified with two DEPC molecules. Above are spectra of identified fragments from mmass and below are tables of mmass results from ESI Q‐TOF MS/MS spectrum, where pink cells are false‐positive results indicated by mmass as DEPC modifications in Aβ1–16 control sample combined with results indicating higher modification level than the precursor. Grey cells are results found by mmass software for copper protected Aβ1–16 modified with two DEPC molecules. ‘0 DEPC’ row indicates peptide fragments without modifications and ‘1–5 DEPC’ indicates the number of modifications found by mmass software. Samples were in 20 mm ammonium acetate, pH 7.4; precursor was peak 1050.5 m/z (two times DEPC‐modified Aβ1–16 with charge 2+), collision energy 50. [file FEB4-10-1072-s006.tif]

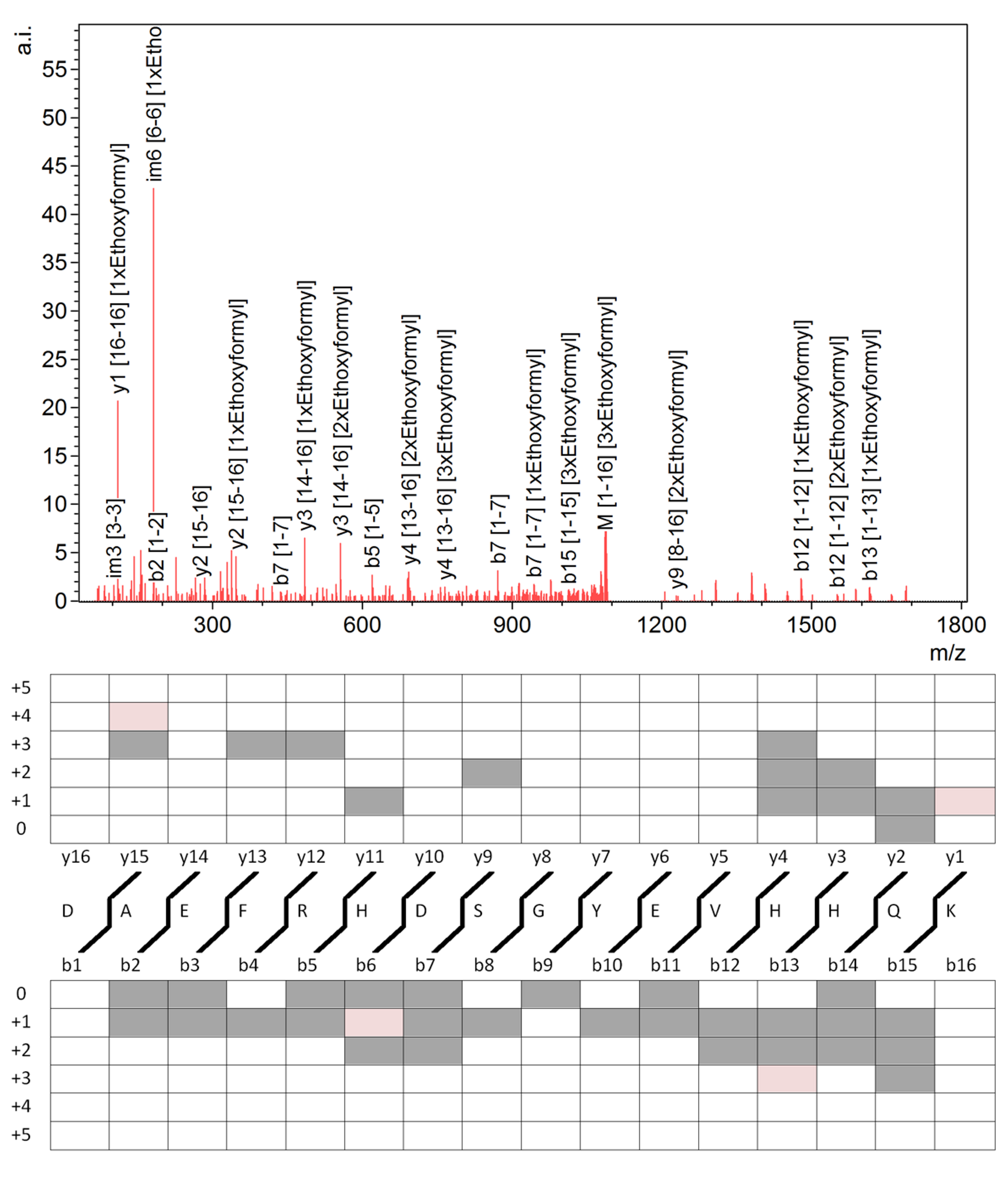

Supplement: Supplementary file 7 — Fig. S7. Targeted sequencing data from the peak of copper protected Aβ1–16 modified with three DEPC molecules. Above are spectra of identified fragments from mmass and below are tables of mmass results from ESI Q‐TOF MS/MS spectrum, where pink cells are false‐positive results indicated by mmass as DEPC modifications in Aβ1–16 control sample combined with results indicating higher modification level than the precursor. Grey cells are results found by mmass software for copper protected Aβ1–16 modified with three DEPC molecules. ‘0 DEPC’ row indicates peptide fragments without modifications and ‘1–5 DEPC’ indicates the number of modifications found by mmass software. Samples were in 20 mm ammonium acetate, pH 7.4; precursor was peak 1086.5 m/z (three times DEPC‐modified Aβ1–16 with charge 2+), collision energy 50. [file FEB4-10-1072-s007.tif]

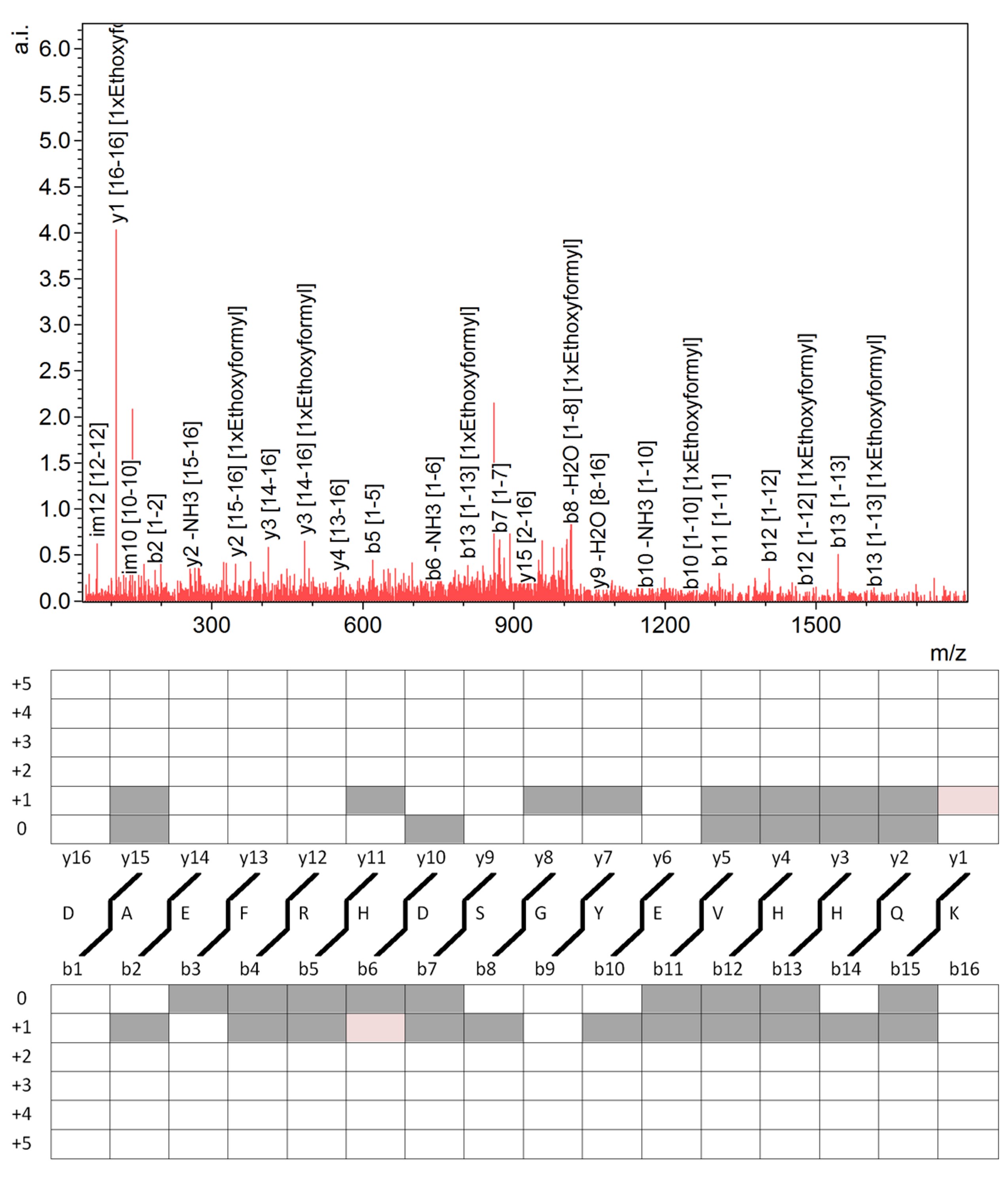

Supplement: Supplementary file 8 — Fig. S8. Targeted sequencing data from the peak of copper protected Aβ1–16 modified with 1 DEPC molecules after hydroxylamine treatment. Above is a spectrum of identified fragments from mmass and below is the table of mmass results from ESI Q‐TOF MS/MS spectrum, where pink cells are false‐positive results indicated by mmass as DEPC modifications in Aβ1–16 control sample combined with results indicating higher modification level than the precursor. Grey cells are results found by mmass software for copper protected Aβ1–16 modified with 1 DEPC molecule. ‘0 DEPC’ row indicates peptide fragments without modifications and ‘1–5 DEPC’ indicates the number of modifications found by mmass software. The sample was in 20 mm ammonium acetate, pH 7.4; precursor was peak 1014 m/z (one‐time DEPC‐modified Aβ1–16 with charge 2+), collision energy 50. [file FEB4-10-1072-s008.tif]
